# Are BCL6 and EZH2 novel therapeutic targets for systemic lupus erythematosus?

**DOI:** 10.1038/s41423-022-00882-1

**Published:** 2022-05-30

**Authors:** Shu Ding, Yu Rao, Qianjin Lu

**Affiliations:** 1 Department of Dermatology, The Third Xiangya Hospital of Central South University, #138 Tongzipo Road, Changsha, 410013 Hunan China; 2 Institute of Dermatology, Chinese Academy of Medical Sciences and Peking Union Medical College, #12 Jiangwangmiao Street, Xuanwu, Nanjing, 210042 Jiangsu China; 3 Key Laboratory of Basic and Translational Research on Immune-Mediated Skin Diseases, Chinese Academy of Medical Sciences, Nanjing, 210042 China

**Keywords:** Autoimmunity, Immunology

As a chronic systemic autoimmune disease, systemic lupus erythematosus (SLE) can influence multiple organs and systems. Although the pathological basis and risk factors for SLE have been extensively investigated, the exact pathogenesis of SLE remains to be explored. However, there is increasing evidence that T-cell−B-cell interactions promote the development of SLE, resulting in the generation of high-affinity antibodies [1]. Tfh cells, a subgroup of T cells, can promote the formation of germinal centers, the maturation of B cells, and the differentiation of plasma cells [2]. It has been found that multiple transcription factors (TFs) can regulate the generation of Tfh cells. For instance, B-cell lymphoma-6 (BCL-6), an evolutionarily conserved zinc-finger protein, is an important positive transcription factor responsible for the differentiation and maturation of Tfh cells. In addition to maintaining the phenotype and GC response of Tfh cells, BCL-6 can also regulate the dynamics of T-cell−B-cell interactions and improve the efficiency of delivering help to B cells [3].

Our previous study investigated the role of BCL-6 in CD4^+^ T cells in SLE and was published in Cellular & Molecular Immunology [4]. Overall, we provided supporting evidence that BCL-6 is a novel factor participating in the pathogenesis of lupus by epigenetically suppressing miR-142-3p/5p expression with the help of EZH2 in CD4^+^ T cells. BCL-6 attaches to the promoter region of miR-142 in CD4^+^ T cells in lupus and recruits EZH2 and HDAC5, which leads to the upregulation of H3K27me3 and the downregulation of H3K9/K14ac, respectively. Consequently, transcription of miR-142 is epigenetically inhibited, and miR-142-3p/5p expression in CD4^+^ T cells in SLE is reduced, which upregulates the expression of CD40L, ICOS and CD84, increases the production of IL-10 and IL-21, and promotes IgG production by B cells.

As BCL-6, IL-21, CD40L and ICOS are all critical regulators involved in the differentiation and maturation of Tfh cells, these findings are helpful for understanding the role that Tfh cells play in lupus. These important data also provide a novel way to therapeutically target BCL-6 and/or certain genes in relevant pathways. Some lupus drugs may inhibit autoantibody production by modulating BCL-6 expression in CD4^+^ T cells. A recent study demonstrated that erythropoietin reduced the expression of BCL-6 in CD4^+^ T cells via a STAT5-dependent mechanism, which directly prevented Tfh cell differentiation and function in vitro and in vivo [5]. Some researchers observed that after treatment with dexamethasone, the IL-21 and BCL-6 expression of CD4^+^ T cells was significantly suppressed, the proportion of Tfh cells was markedly decreased, and the proportion of Tfh cells was positively correlated with the level of autoantibodies in both Balb/c mice and MRL/lpr mice [6]. Moreover, the importance of the interaction between BCL-6 and IL-21 in Tfh cell differentiation and autoantibody production should not be ignored, especially in lupus and some autoimmune diseases. We demonstrated that the overexpression of BCL-6 in CD4^+^ T cells in lupus decreased the expression of miR-142-3p/5p, and inhibition of miR-142-3P/5P, and led to increased expression of IL-21 [4]. Huang et al. reported that a subpopulation of CD4^+^CXCR5^hi^PD-1^hi^BCL-6^+^ circulating Tfh cells, rather than a population of CD4^+^CXCR5^hi^PD-1^hi^ cells, was positively correlated with SLEDAI and anti-dsDNA antibody levels in the cTfh subpopulation of SLE patients. Furthermore, elevated production of IL-21 can greatly upregulate the transcription levels of BCL-6 by enriching TET2 on the promoter of BCL-6 in CD4^+^ T cells in SLE [7]. Ricard et al. found that patients with systemic sclerosis presented an activated Tfh cell phenotype, with upregulated expression of BCL-6, which contributed to an increase in the production of IL-21 [8]. A more recent study on gene expression confirmed the upregulated expression of BCL-6 and genes regulated by BCL-6 in CD4^+^ T cells in granulomatosis with polyangiitis. When CD4^+^ T cells in granulomatosis with polyangiitis were activated in vitro, BCL-6 expression was sustained, and Tfh cell differentiation resulted in significant increases in IL-21 and IL-6 production [9]. All this evidence suggests that the positive feedback loop regulating BCL-6-IL-21 may be important for the differentiation and maturation of Tfh cells. Moreover, Tfh cells play a significant role in the occurrence of SLE, so we can conclude that the positive feedback loop regulation of BCL-6-IL-21 may promote Tfh cell differentiation and function in CD4 + T cells in lupus and prompt the interaction of T cells and B cells, resulting in overproduction of self-reactive antibodies and thus promoting the occurrence and development of SLE. Based on the above findings, BCL-6 may be a biological therapeutic target with high specificity for lupus.

It has been identified that epigenetic mechanisms are involved in BCL-6 regulation. Previous studies have reported that DNA methylation, histone modification and miRNAs all take part in the upstream and downstream regulation of BCL-6. EZH2, which is the central component of polycomb-repressive complex 2 (PRC2), participates in cell survival, differentiation, migration, proliferation, etc. EZH2 is a chromatin-modifying enzyme that can promote the methylation of histone H3 at lysine 27 and has been predominantly studied for its role in various epigenetic modifications. A new mechanism revealed by us indicates that BCL-6 recruits EZH2 to the promoter of miR-142 in CD4 + T cells in lupus to increase the expression of miR-142-3p/5p [4]. A recent study in BioFactors confirmed the cooperation of BCL-6 and EZH2. The researchers found that BCL-6 increased the H3K27me3 level in the miR-34a promoter via EZH2 recruitment in cardiomyocytes, which inhibited the apoptosis of myocardial cells [10]. Given the effect of the cooperation of BCL-6 and EZH2 on miR-142-3p/5p expression in CD4^+^ T cells in lupus, we wondered whether EZH2 could be a therapeutic target for lupus treatment. Another study by our team proved that UHRF1 downregulation leads to decreased DNA methylation and H3K27me3 levels in the BCL-6 gene promoter region, promoting Tfh cell differentiation in SLE; this effect is probably a result of the interaction of UHRF1 with DNMT1 and EZH2, which forms a regulatory complex of gene promoters [11]. E4BP4 led to a decreased H3 acetylation level but also to an increased H3K27me3 level in the BCL-6 gene promoter region by recruiting the repressive epigenetic modifiers HDAC1 and EZH2. E4BP4 deficiency in CD4+ T cells in lupus resulted in overexpression of BCL-6, which promoted Tfh cell differentiation and function and exacerbated pristane-induced lupus-like diseases [12]. This finding indicates that EZH2 may cooperate with other transcription factors to regulate BCL-6 expression in CD4^+^ T cells in lupus. Studies on other diseases have shown similar findings. H3K27me3 modification by EZH2 is an important factor that positively affects BCL-6 expression, which modulates the differentiation of early Tfh cells during acute viral infection [13]. Moreover, EZH2 makes regulates BCL-6 expression in various ways in addition to changing the level of H3K27me3 in the BCL-6 gene promoter. Most EZH2 bound to areas of the BCL-6 promoter in Tfh cells is associated with H3K27ac, and fewer gene loci include H3K27me3 modifications conferred by EZH2. After EZH2 phosphorylation at Ser21, EZH2 can be recruited by TCF1 to directly activate BCL-6 transcription [14]. Generally, BCL-6 can cooperate with EZH2 to regulate the transcription of downstream genes by regulating H3K27me3 levels, while the cooperation of EZH2 with other transcription factors can not only regulate the transcription of BCL-6 by regulating H3K27me3 levels but also directly activate the transcription of BCL-6. Although research has indicated that, compared with healthy controls, CD4^+^ T cells in lupus have increased expression of EZH2 [15], the biological effects of EZH2 depended more on EZH2 protein modification and the interaction of EZH2 with other functional proteins than on the EZH2 expression level in cells. Therefore, EZH2 may not be suitable as a target for SLE treatment. Of course, more research should be carried out in this area.

**Fig. 1:**
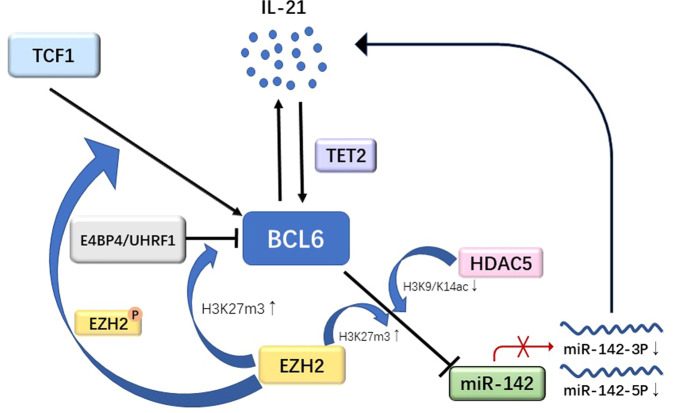
The roles of BCL-6 and EZH2 in CD4+ T cells. BCL-6 can inhibit miR-142-3p/5p expression by recruiting EZH2 and HDAC5 to the miR-142 promoter, which results in the upregulation of H3K27me3 and the downregulation of H3K9/K14ac, and inhibition of miR-142-3P/5P increases IL-21 production. IL-21 upregulates the transcription levels of BCL-6 by increasing TET2 levels in the promoter of BCL-6. E4BP4 or UHRF1 increase H3K27me3 levels in the promoter of BCL-6 by recruiting EZH2. After EZH2 phosphorylation at Ser21, EZH2 can be recruited by TCF1 to directly activate BCL-6 transcription

In conclusion, the results of existing studies have improved our understanding of the novel pathogenic roles of BCL-6 and EZH2 in SLE (Fig. 1). Nevertheless, the study of regulatory networks involving BCL-6 and EZH2 in lupus remains a major challenge at the genome level. By elucidating the functions and interactions of these genes, it will be possible to further determine how Tfh cells stimulate the production of self-reactive antibodies by B cells, which could help clarify the molecular pathophysiology of SLE and even provide targeted therapeutic strategies for lupus and other autoimmune diseases.
